# Joint Association of Occupational and Leisure‐Time Physical Activity With Low Back Pain in Korean Adults

**DOI:** 10.1002/msc.70096

**Published:** 2025-04-04

**Authors:** Sanghun Yim, DooYong Park

**Affiliations:** ^1^ Department of Special Physical Education Yongin University Yongin Korea; ^2^ Department of Physical Education Seoul National University Seoul Korea

**Keywords:** aged, cohort studies, epidemiology, low back pain, physical activity

## Abstract

**Objective:**

This study aimed to examine the effect of the interaction between gender‐specific occupational physical activity (OPA) and leisure‐time physical activity (LTPA) on low back pain (LBP) risk.

**Methods:**

Data were obtained from a large‐scale cohort survey of Koreans, comprising a total of 2750 participants recruited during 2011–2012. OPA and LTPA were assessed using validated questionnaires and classified into two groups: “< 150 min/wk” and “≥ 150 min/wk.” LBP was defined based on the Oswestry Disability Index (ODI) and visual analogue scale (VAS) criteria. Multiple logistic regression analyses were performed to calculate odds ratios (OR) with 95% confidence intervals (CI), and multiple linear regression analyses were used to estimate *β* coefficients with 95% CI to assess the associations between OPA, LTPA, and LBP.

**Results:**

The results showed that increased OPA was significantly associated with higher ODI scores (*β* = 0.02, *p* = 0.021), whereas increased LTPA was significantly associated with reductions in both ODI (*β* = −0.07, *p* = 0.012) and VAS scores (*β* = −0.01, *p* = 0.013). Furthermore, engaging in OPA for ≥ 150 min/week was associated with an elevated LBP risk (OR = 1.38, 95% CI = 1.12–1.69), with significant gender differences observed. In contrast, for participants with < 150 min/wk of leisure‐related physical activity, LBP risk increased (OR = 1.22, 95% CI = 1.01–1.76), whereas for those with ≥ 150 min/wk, LBP risk decreased (OR = 0.84, 95% CI = 0.66–0.95), a significant effect observed only in females.

**Conclusion:**

These findings suggest that ensuring adequate rest during OPA and promoting leisure‐related physical activity may be critical strategies for reducing chronic LBP.

## 1 Introduction

Low back pain (LBP) refers to a condition where pain persists in the lower back for 3 months or more (Wells et al. 2014), and individuals with chronic LBP experience reduced ability to perform daily activities, often resulting in disability (Smith and Osborn 2007). Among the causes of LBP, approximately 85% remain unexplained (Goertz et al. 2012). As a result, LBP significantly impacts the quality of life (Jonsdottir et al. 2019), and is a major social, clinical, and economic issue worldwide (Henschke et al. 2015). According to the 2021 Global Burden of Disease study, which tracked the prevalence of LBP from 1990 to 2021, there were 628.8 million cases of LBP globally, with a higher prevalence among females than males. The study also indicated that the incidence of new LBP cases continues to rise each year (Cheng et al. 2025). In South Korea, the 2025 National Health Insurance Service report revealed that the medical expenses related to lumbar and pelvic pain totalled 366.1 billion KRW in 2023, contributing to the country's 110 trillion KRW in total health insurance expenditures (Health Insurance Review and Assessment Service 2025). Consequently, LBP presents both a significant global health burden and a public health priority, with high prevalence rates and associated economic costs (Cheng et al. 2025).

It is well established that increased physical activity(PA) is an important factor in reducing LBP (Gordon and Bloxham 2016), and studies have shown a strong association between PA and physical function in older adults, with more active individuals being better able to recover from both acute and chronic LBP (Cedraschi et al. 2016; Koes et al. 2006). While numerous studies have explored the relationship between PA and LBP risk, findings have varied based on the type of PA. Specifically, occupational physical activity(OPA) has been linked to an increased risk of LBP, while leisure‐time physical activity(LTPA) has been associated with a reduction in both the incidence and severity of LBP (Heneweer et al. 2011).

However, most studies focus on the relationship between increased LTPA and reduced LBP incidence, with little exploration of the differential effects of leisure‐time versus OPA(Gordon and Bloxham 2016; Shnayderman and Katz‐Leurer 2013). Although some research has examined the association between OPA and LTPA and LBP risk (Anitha et al. 2019; Jung et al. 2024), these studies have not addressed gender differences or the interaction between occupational and leisure‐time activities on LBP risk (A et al., 2019). Furthermore, a study suggesting that increased LTPA can mitigate the risk of LBP caused by occupational activity focused solely on commuting time and did not investigate how different types of PA influence LBP severity. This highlights the need for future research to utilise validated surveys to assess the relationship between specific types of PA and LBP (Jung et al. 2024).

Therefore, this study aims to examine the changes in the LBP disability index and LBP intensity related to LBP according to the amount of time spent in different types of PA. We also aim to explore the relationship between gender, PA type, and LBP risk. Additionally, this study will investigate the interaction between OPA, LTPA and its impact on LBP risk.

## 2 Methods

### 2.1 Study Participants

This study utilised data from the Korean Genome and Epidemiology Study (KoGES), a large cohort investigation aimed at preventing chronic disease. Participants were general adults aged 49–79 years residing in Ansan City, Gyeonggi Province, who participated in the 2011–2012 KoGES survey. A total of 3052 individuals were recruited via phone, mail, and home visits. After excluding 302 participants with missing data on variables related to LBP and PA participation time (gender, age, sleep duration, smoking status, alcohol consumption, income level, occupation, obesity level, inflammation level, muscle mass, and daily sitting time), a total of 2750 participants were included in the analysis. All participants provided written informed consent after being informed about the purpose and procedures of the study.

### 2.2 Measurement Variables

#### 2.2.1 LBP and VAS

LBP was measured using the Korean version of the Oswestry Disability Index (ODI), which consists of 10 domains: LBP intensity, personal care, lifting, walking, sitting, standing, sleeping, sexual activity, social life, and travel. The total score was calculated by summing the scores from each item, dividing by the maximum possible score, and multiplying by 100 to yield a percentage(%) (Brokelman et al. 2012). ODI has demonstrated high test‐retest reliability (0.93) and internal consistency (0.92) in assessing lumbar disability.

The intensity of LBP was measured using the Visual Analogue Scale (VAS), where participants indicated their LBP intensity on a 10 cm scale, with 0 indicating no pain and 10 indicating the most severe pain. The test‐retest reliability of VAS was 0.95 (Brokelman et al. 2012). LBP was classified as “LBP” if participants reported severe LBP within the past 6 months, had an ODI score of 20% or higher, or a VAS score of 4 or higher, based on previous studies (Brokelman et al. 2012; Williams and Johnson 2024). Those not meeting these criteria were classified as “non‐LBP.”

#### 2.2.2 OPA and LTPA

PA was assessed using the International Physical Activity Questionnaire – Long Form (IPAQ‐LF), which includes 27 questions and allows a more detailed assessment of PA participation compared to the short form. This tool has been shown to predict disease prevalence more accurately (International Physical Activity Questionnaire Research Committee 2005). OPA and LTPA levels were categorised based on previous studies, where participation of less than 150 min per week was associated with increased risk of disease. Therefore, the classification was as follows: “≤ 150 min/week” and “≥ 150 min/week” (Cho et al. 2021).

#### 2.2.3 Other Variables

Surveys were conducted through one‐on‐one interviews by trained surveyors, followed by review and corrections to ensure completeness. Body measurements, including height (cm) and weight (kg) were recorded. Height and weight were measured once with the value used for analysis. Body mass index (BMI) was calculated as weight (kg) divided by height squared (m^2^). Muscle mass was measured using a Zeus 9.9 (JAWON Medical, Korea) device. Alcohol consumption was classified as “no alcohol experience,” “past drinking,” or “current drinking” based on responses to questions about alcohol use. Smoking status was classified as “no smoking experience,” “past smoking,” or “current smoking” based on responses to questions about smoking history. Sleep duration was calculated as the average of weekday and weekend sleep hours using the formula ((weekday sleep hours × 5) + (weekend sleep hours × 2))/7 and was used as a continuous variable. Family income was categorised as “< 1 million KRW,” “1–2 million KRW,” “2–3 million KRW,” “3–4 million KRW,” “4–6 million KRW,” and “≥ 6 million KRW.” Occupation was categorised into “professional/managerial/clerical,” “sales/service workers,” “agriculture, forestry, fisheries, and other technical occupations,” “household workers,” and “others” based on occupation classification codes. High‐sensitivity C‐reactive protein (hs‐CRP) was measured from blood samples collected after 8 h of fasting, processed locally with a centrifuge, and analysed using an ADVIA 1800 auto analyser (Siemens, USA) at Seoul Clinical Laboratory. Total sitting time was calculated using responses to questions about weekday and weekend sitting time using the formula ((weekday sitting time × 5) + (weekend sitting time × 2))/7 and was used for analysis.

### 2.3 Statistical Analysis

Data analysis was conducted using STATA/IC 14.1 (STATA Corp., College Station, TX, USA). Descriptive analysis of the demographic characteristics of the participants was performed using frequency analysis and mean calculations via chi‐square tests and *t*‐tests. Each variable was expressed as percentages or means and standard deviations (Tables 1 and 2). To examine the changes in ODI scores and VAS scores associated with PA participation time, linear regression analysis was used to estimate *β* values and 95% confidence intervals (95% CI) (Table 3).

**TABLE 1:** Baseline characteristics of the subjects by gender.

Characteristics of risk factors	Total	Male	Female	*p*
	(*n* = 2750)	(*n* = 1465)	(*n* = 1285)	
LBP (%)	42.69	38.50	47.47	< 0.001
Age (yr)	57.85 ± 7.01	58.02 ± 7.03	57.65 ± 6.99	0.171
ODI score	12.56 ± 14.86	11.77 ± 14.00	13.46 ± 15.75	0.003
VAS score	2.12 ± 2.74	1.76 ± 2.51	2.51 ± 2.93	< 0.001
BMI (kg/m^2^)	24.74 ± 2.93	24.82 ± 2.74	24.64 ± 3.12	0.094
Lean body mass (kg)	44.64 ± 8.19	50.71 ± 5.76	37.73 ± 3.99	< 0.001
hs‐CRP (mg/dL)	1.42 ± 3.38	1.51 ± 3.31	1.32 ± 3.46	0.156
Current drinking (%)	48.91	69.90	24.98	< 0.001
Current smoking (%)	13.53	24.30	1.25	< 0.001
Low income (%)	11.02	8.33	14.09	< 0.001
Sleep time (hr/day)	6.14 ± 1.19	6.29 ± 1.15	5.96 ± 1.21	< 0.001
Daily sitting time (hr/day)	5.74 ± 2.64	5.86 ± 2.88	5.61 ± 2.34	0.013
OPA (min/wk)	252.71 ± 727.61	314.93 ± 793.96	181.76 ± 636.60	< 0.001
LTPA (min/wk)	245.91 ± 298.92	273.21 ± 33.47	214.78 ± 247.33	< 0.001
Classification of occupations (%)				
Managers, professionals, clerical worker	28.44	42.18	12.76	< 0.001
Services and sales worker	10.44	6.62	14.79	
Agricultural, forestry, fishery worker and technicians	15.71	27.51	2.26	
Homemaker	30.33	0.00	64.90	
Et cetera	15.09	23.69	5.29	

*Note:* Continuous data are presented as means (mean ± SD) and categorical data are presented as %.

Abbreviations: BMI, body mass index; hs‐CRP, high sensitivity‐C reactive protein; LBP, low back pain; LTPA, leisure‐time physical activity; ODI, oswestry disability index; OPA, occupational physical activity; SD, standard deviation; VAS, visual analogue scale.

**TABLE 2:** Baseline characteristics of the subjects by different types of physical activities.

Characteristics of risk factors	Total (*n* = 2750)					
	OPA			LTPA		
	< 150 min/wk (*n* = 2181)	≥ 150 min/wk (*n* = 569)	*p*	< 150 min/wk (*n* = 1292)	≥ 150 min/wk (*n* = 1458)	*p*
LBP variable						
ODI score	12.27 ± 14.97	13.65 ± 14.38	0.048	13.30 ± 15.10	11.90 ± 14.62	0.014
VAS score	2.11 ± 2.77	2.12 ± 2.65	0.966	2.27 ± 2.80	1.98 ± 2.69	0.006
LBP (%)	41.63	46.75	0.028	45.59	40.12	0.004
						
Daily activity variable						
Sleep time (hr/day)	6.12 ± 1.20	6.19 ± 1.13	0.196	6.10 ± 1.20	6.17 ± 1.18	0.146
Daily sitting time (hr/day)	6.03 ± 2.66	4.63 ± 2.25	< 0.001	5.79 ± 2.73	5.70 ± 2.57	0.361
						
Other variable						
BMI (kg/m^2^)	24.71 ± 2.94	24.82 ± 2.86	0.433	24.81 ± 3.05	24.67 ± 2.81	0.201
Lean body mass (kg)	43.92 ± 8.13	47.40 ± 7.82	0.001	44.10 ± 8.16	45.12 ± 8.19	0.001
hs‐CRP (mg/dL)	1.44 ± 3.54	1.35 ± 2.68	0.573	1.42 ± 2.66	1.42 ± 3.91	0.981

*Note:* Continuous data are presented as means (mean ± SD) and categorical data are presented as %.

Abbreviations: BMI, body mass index; hs‐CRP, high sensitivity‐C reactive protein; LBP, low back pain; LTPA, leisure‐time physical activity; ODI, oswestry disability index; OPA, occupational physical activity; SD, standard deviation; VAS, visual analogue scale.

**TABLE 3:** Relationship between types of physical activity and LBP‐ VAS score and LBP‐ODI score.

Characteristics of risk factors	ODI score (*n* = 2750)			VAS score (*n* = 2750)		
	β	SE	*p*	β	SE	*p*
OPA (per 30 min/wk)	0.02	0.01	0.021	0.01	0.01	0.048
LTPA (per 30 min/wk)	−0.07	0.02	0.012	−0.01	0.01	0.013
Work & LTPA (per 30 min/wk)	0.01	0.01	0.238	0.01	0.01	0.376

*Note:* The regression *β* coefficient (*β*) with its standard error (SE) and the *p*‐value are given for each association. Multiple linear regression models adjusted age, gender, income level, sleep duration, alcohol consumption, current smoking, hs‐CRP, classification of occupations, daily sitting time, lean body mass, BMI.

Abbreviations: BMI, body mass index; hs‐CRP, high sensitivity‐C reactive protein; LBP, low back pain; LTPA, leisure‐time physical activity; ODI, oswestry disability index; OPA, occupational physical activity; VAS, visual analogue scale.

Logistic regression analysis was used to assess the association between PA participation time by type and LBP risk, calculating odds ratios (ORs) and 95% CI (Table 4). The interaction between OPA and LTPA and LBP risk was also analysed using the same model (Table 5). Confounding variables such as age, gender, sleep duration, hs‐CRP, alcohol consumption, smoking status, income level, occupation type, obesity level, muscle mass, and daily sitting time were adjusted for in all analyses.

**TABLE 4:** Odds ratio of LBP risk according to types of physical activities.

Characteristics of risk factors	Total (*n* = 2750)	Male (*n* = 1465)	Female (*n* = 1280)
OPA			
< 150 min/wk	1.00 (reference)	1.00 (reference)	1.00 (reference)
≥ 150 min/wk	1.38** (1.12, 1.69)	1.31^a^ (1.02, 1.70)	1.44 (0.99, 2.08)
			
LTPA			
< 150 min/wk	1.00 (reference)	1.00 (reference)	1.00 (reference)
≥ 150 min/wk	0.79** (0.67, 0.93)	0.86 (0.69, 1.07)	0.75^a^ (0.59, 0.94)
			
Work &LTPA			
< 150 min/wk	1.00 (reference)	1.00 (reference)	1.00 (reference)
≥ 150 min/wk	0.96 (0.79, 1.16)	1.09 (0.81, 1.47)	0.90 (0.69, 1.17)

*Note:* Model 1 adjusted age, gender, income level, sleep duration, alcohol consumption, current smoking, hs‐CRP, classification of occupations, daily sitting time, lean body mass, BMI. Model 2 adjusted model1 plus weekly LTPA.

Abbreviations: BMI, body mass index; hs‐CRP, high sensitivity‐C reactive protein; LBP, low back pain; low back pain; LTPA, leisure‐time physical activity; OPA, occupational physical activity.

^a^ *p* < 0.05, **p < 0.01, ***p < 0.001.

**TABLE 5:** Joint association of OPA and LTPA with LBP risk.

Characteristics of risk factors	Total (*n* = 2750)	Male (*n* = 1465)	Female (*n* = 1280)
OPA x LTPA			
Both < 150 min/wk	1.00 (reference)	1.00 (reference)	1.00 (reference)
OPA ≥ 150 min/wk & LTPA < 150 min/wk	1.22^a^ (1.01, 1.76)	1.22 (0.86, 1.74)	1.57 (0.97, 2.54)
OPA < 150 min/wk & LTPA ≥ 150 min/wk	0.84^a^ (0.66, 0.95)	0.84 (0.65, 1.10)	0.76^a^ (0.59, 0.97)
Both ≥ 150 min/wk	1.17 (0.83, 1.48)	1.17 (0.81, 1.69)	1.03 (0.62, 1.71)

*Note:* Model 1 adjusted age, gender, income level, sleep duration, alcohol consumption, current smoking, hs‐CRP, classification of occupations, daily sitting time, lean body mass, BMI. Model 2 adjusted model1 plus weekly LTPA.

Abbreviations: BMI, body mass index; hs‐CRP, high sensitivity‐C reactive protein; LBP, low back pain; low back pain; LTPA, leisure‐time physical activity; OPA, occupational physical activity.

^a^ *p* < 0.05, **p < 0.01, ***p < 0.001.

## 3 Results

The demographic characteristics by gender in this study are presented in Table 1. Compared to female, male exhibited a lower prevalence of LBP, lower ODI scores, lower VAS scores, and lower proportions of low‐income individuals and homemakers, while showing higher muscle mass, smoking rates, alcohol consumption rates, sleep duration, total daily sitting time, OPA participation time, LTPA participation time, and higher proportions of managers, professionals, and clerical workers.

Additionally, the demographic characteristics by PA type are presented in Table 2. For individuals engaging in OPA for at least 150 min per week, compared with those participating for less than 150 min, daily sitting time was lower, and ODI scores, LBP prevalence, and muscle mass were higher. In contrast, for LTPA, those participating for at least 150 min per week had lower ODI scores, lower VAS scores, and a lower LBP prevalence, along with higher muscle mass, compared with those with less than 150 min of participation.

Table 3 displays the associations between PA participation time and VAS and ODI scores. With each additional 30 min per week of OPA, the ODI score increased by 0.02 points (*p* = 0.021); conversely, each additional 30 min per week of LTPA was associated with significant decreases in both ODI (*β* = −0.07, *p* = 0.012) and VAS scores (*β* = −0.01, *p* = 0.013).

The independent associations between PA participation time by type and LBP risk, stratified by gender, are presented in Table 4. After adjusting for various confounders, the group engaging in OPA for at least 150 min per week had a 1.38‐fold higher LBP risk (OR = 1.38, 95% CI = 1.12–1.69) compared with the group with less than 150 min of participation; this effect was observed only among men (OR = 1.31, 95% CI = 1.02–1.70). Conversely, for LTPA, the group with at least 150 min per week demonstrated a 21% reduction in LBP risk (OR = 0.79, 95% CI = 0.67–0.93) compared with those with less than 150 min; this association was significant only among women (OR = 0.75, 95% CI = 0.59–0.94). When the combined total participation time in OPA and LTPA was at least 150 min per week, no significant association with LBP risk was observed (*p* > 0.05).

Table 5 presents the interaction between OPA and LTPA by gender in relation to LBP risk. Compared to the group that participated in all physical activities for less than 150 min per week, the group with OPA of at least 150 min per week and LTPA of less than 150 min per week exhibited a 1.22‐fold increase in LBP risk (OR = 1.22, 95% CI = 1.01–1.76). In contrast, the group with OPA of less than 150 min per week and LTPA of at least 150 min per week showed a 16% reduction in LBP risk (OR = 0.84, 95% CI = 0.66–0.95). Moreover, among women, compared to the group with less than 150 min of PA in all categories, the combination of OPA for less than 150 min per week with LTPA for at least 150 min per week was significantly associated with a reduced LBP risk (OR = 0.76, 95% CI = 0.59–0.97); no significant associations were observed among men(*p* > 0.05).

## 4 Discussion

This study found that increased participation in OPA was associated with higher ODI scores, whereas increased LTPA was associated with reductions in both ODI and VAS scores. Moreover, engaging in OPA for at least 150 min per week was linked to an increased OR for LBP among the overall population and among males, while engaging in LTPA for at least 150 min per week was associated with a decreased LBP risk among the overall population and among females. In addition, groups with OPA of at least 150 min per week combined with LTPA of less than 150 min per week exhibited an increased LBP risk, whereas groups with OPA of less than 150 min per week combined with LTPA of at least 150 min per week demonstrated a decreased LBP risk, with this association reaching significance only among females.

Previous research suggests that the relationship between PA and LBP is complex, emphasising that both excessive and insufficient PA may contribute to the development of LBP (Wai et al. 2008). Prior studies have reported that increased time spent on heavy lifting can raise the risk of LBP up to fivefold (OR = 4.99, 95% CI = 1.33–18.74), whereas increased LTPA reduces LBP risk by 56% (OR = 0.56, 95% CI = 0.44–0.91) (A et al., 2019). Furthermore, in studies involving chronic LBP patients, a 6‐week aerobic exercise programme resulted in a 20% reduction in pain, while a strength training programme yielded a 15% reduction (Shnayderman and Katz‐Leurer 2013). These findings confirm that OPA and LTPA exert different effects on LBP.

The positive association between OPA and LBP risk observed in Tables 3, 4, 5 may be explained by previous studies showing that high‐intensity OPA is closely related to musculoskeletal pain due to fatigue (Roquelaure et al. 2012). In our study, as shown in Table 2, individuals engaging in OPA for at least 150 min per week had significantly lower daily sitting time (6.03 h/day vs. 4.63 h/day) yet a significantly higher LBP prevalence, which is consistent with prior findings. Unlike LTPA, occupational activity often does not allow sufficient rest, and the high loads and repetitive stress associated with such activities may induce LBP (Krause et al. 2015; Yilmaz and Dedeli 2012). Therefore, the lower sitting time observed in the group with ≥ 150 min of OPA may indicate insufficient rest, and the repetitive nature of manual labour likely contributes to increased fatigue and, consequently, a higher LBP risk (Heneweer et al. 2011; Roquelaure et al. 2012).

Conversely, the inverse association between LTPA and LBP risk, as seen in Tables 3, 4, 5, may be attributed to the role of core muscle strength. Previous research indicates that weakened abdominal muscles are strongly associated with LBP (Simon and Hicks 2018), and diminished core strength reduces lumbar stability, thereby increasing the likelihood of LBP (Danneels et al. 2000). Studies have also shown that strength training and walking activate core muscles and improve functional performance in LBP patients (Shnayderman and Katz‐Leurer 2013). Thus, strengthening deep abdominal muscles through LTPA is essential for supporting the lumbar spine and may help reduce LBP (Goertz et al. 2012; Koumantakis et al. 2005).

The significant association between LTPA and lower VAS scores observed in Table 3 may be due to the fact that aerobic PA stimulates the production of endorphins, which serve as a non‐pharmacological means of reducing perceived pain (Gordon and Bloxham 2016). Therefore, participation in at least 150 min of LTPA per week may reduce LBP intensity through enhanced endorphin production (Gordon and Bloxham 2016; Kenney et al. 2022).

The significant association between OPA participation time and LBP risk among males, as shown in Table 4, may be explained by the fact that, according to Table 1, males engage in OPA for 1.73 times longer than females (314.93 min/wk vs. 181.76 min/wk). This suggests that males are more frequently exposed to occupational activities involving bending and twisting, which negatively impact the lower back and increase LBP risk (Heneweer et al. 2011).

For females, the significant inverse association between LTPA participation time and LBP risk observed in Tables 4 and 5 may be explained by the higher prevalence of LBP (47.47% vs. 38.50%) and a greater proportion of homemakers (64.90% vs. 0.00%) compared to males, which is consistent with previous findings linking higher LBP rates in females to increased engagement in household activities that involve repetitive upper‐body bending (Habib et al. 2012). Furthermore, females engaged in LTPA for 58 min less per week (214.78 min/wk vs. 273.21 min/wk), suggesting that lower leisure activity levels may contribute to decreased core muscle strength and, consequently, higher LBP prevalence (Marijančić et al. 2024; Zorlular et al. 2022). Thus, for females, engaging in at least 150 min of LTPA may help strengthen weakened lower back muscles, thereby reducing LBP risk (Amit et al. 2013).

In Table 5, the absence of a significant association between overall participation (combining OPA and LTPA of at least 150 min per week) and LBP risk may be due to the offsetting positive effects of LTPA (Simon and Hicks 2018) and negative effects of OPA (Heneweer et al. 2011) on LBP.

This study is significant in that it differentiates PA into occupational and leisure‐related types and examines their independent associations with LBP risk. However, several limitations exist. First, PA type was assessed via self‐report questionnaires, which may be subject to recall bias. Future studies should combine accelerometer‐based PA measurements with time‐specific records of activity type to provide more objective data. Second, this study measured only total muscle mass, which precluded the evaluation of abdominal muscle mass—a key factor directly influencing LBP. Future research should assess LBP risk while adjusting for abdominal muscle mass. Third, as a cross‐sectional study, this research cannot definitively establish causality between the independent and dependent variables. Nonetheless, given the large sample size drawn from national data, the findings serve as valuable baseline information for future longitudinal investigations.

## 5 Conclusion

In this study, participation in OPA was associated with increased LBP disability index scores, whereas LTPA was linked to reductions in both the LBP disability index and LBP intensity. Moreover, the duration of PA participation exhibited distinct associations with LBP risk, with notable gender differences observed. In conclusion, engagement in OPA may exacerbate LBP, suggesting that increasing LTPA could help mitigate these adverse effects. Therefore, to reduce chronic LBP, it is essential to ensure adequate rest during OPA and to promote national strategies that enhance LTPA for LBP relief.

## Author Contributions

Study concept and design: DooYong Park, acquisition of data: DooYong Park, analysis and interpretation of data: DooYong Park, Sanghun Yim, draughting of the manuscript: DooYong Park and Sanghun Yim, critical revision of the manuscript: Sanghun Yim, administrative, technical, or material support: Sanghun Yim, statistical analysis: DooYong Park, Sanghun Yim, and study supervision DooYong Park.

## Ethics Statement

This study was approved by the Institutional Review Board (IRB) of Korea University Ansan Hospital and the Seoul National University Bioethics Committee (IRB No. E2501/004‐006). All participants (Sanghun Yim, DooYong Park) provided written informed consent prior to enrolment, and all study procedures were conducted in accordance with the Declaration of Helsinki and relevant local regulations.

## Conflicts of Interest

The authors (DooYong Park, Sanghun Yim) declare that the research was conducted in the absence of any commercial or financial relationships that could be construed as a potential conflict of interest.

## Data Availability

The data that support the findings of this study are available on request from the corresponding author. The data are not publicly available due to privacy or ethical restrictions.
